# Health facility-based counselling and community outreach are associated with maternal dietary practices in a cross-sectional study from Tanzania

**DOI:** 10.1186/s40795-021-00447-x

**Published:** 2021-08-20

**Authors:** Kirk A. Dearden, Ramu Bishwakarma, Benjamin T. Crookston, Benesta T. Masau, Generose I. Mulokozi

**Affiliations:** 1 IMA World Health/Corus International, Washington DC, USA; 2 Lutheran World Relief/Corus International, Baltimore, MD USA; 3 Department of Health Science, Brigham Young University, Provo, UT USA; 4 IMA World Health, Dar es Salaam, Tanzania

**Keywords:** Maternal, Dietary practices, Tanzania, Counselling, Interpersonal communication, Community health worker, Support groups

## Abstract

**Background:**

Anemia and underweight among women are major public health challenges. Access to health services can improve dietary behaviors and women’s nutritional status. We examined whether exposure to health services is associated with women’s dietary practices in Tanzania.

**Methods:**

Data come from a cross-sectional baseline survey among 5000 female primary caregivers who were randomly selected via two-stage sampling, prior to implementing a maternal and child nutrition program. We ran frequencies on women’s exposure to existing health facility-based counselling, community health worker visits, and attendance at women’s support groups. We examined associations between exposure to these interventions and maternal diets and adjusted for sociodemographic covariates using ordinary least squares regression and ordered logistic regression.

**Results:**

A third of the sample (34.1%) had received any antenatal care (ANC) during their most recent pregnancy or had been advised by anyone about nutrition (37.0%). 68.0% had never had a community health worker (CHW) speak to them about their children’s health and 9.4% had participated in a women’s group. Only 8.0% of mothers ate more than usual during pregnancy and 7.1% ate more types of foods. After adjusting for mother’s age, education and household assets, women who received nutrition advice were 1.3 times (95% CI: 1.1, 1.7) more likely than mothers who did not to eat more during pregnancy. Receiving antenatal care (ANC) and advice on nutrition before, during, and after pregnancy and delivery were highly associated with the mother eating more types of foods. Hearing from a CHW about children’s health but not support group attendance was often associated with various dietary practices. Almost all measures of access to health services were significantly associated with mothers’ frequency of eating in the previous 24 h. Receiving advice on nutrition during pregnancy and after giving birth and CHW contact were associated with mothers’ dietary diversity in the previous 24 h.

**Conclusions:**

Several program exposure variables—especially being counselled about nutrition—were associated with improved dietary practices. Improving service delivery at scale may contribute to improved dietary behaviors in larger populations, given the associations we describe, along with findings from the existing literature.

## Background

While the prevalence of anemia among nonpregnant women of reproductive age has decreased from 43% in 1995 to 38% in 2011 [1], only a few countries are on track to meet the World Health Assembly target of a 50% reduction by 2025 [2]. Globally, 39% of all women of reproductive age are anemic, as are 46% of pregnant women in developing countries [3] with about one in 10 (9.7%) being underweight [2]. Anemia and underweight have long-term consequences for women and children, including increased maternal and child morbidity and mortality and impaired mental development [4–6].

Despite improvements in maternal and child health over the past 20 years, malnutrition in Tanzania remains a major challenge with 53% of pregnant women experiencing anemia (hemoglobin concentration < 11 g/dl), 10% of women of reproductive age suffering from underweight (body mass index or BMI < 18.5 kg/m2), 18.4% overweight (BMI 25- < 30 kg/m2), and 10.0% obese (body mass index > = 30 kg/m2) [7].

Inadequate dietary intake is a major source of malnutrition; [4, 6], in Tanzania, according to one study, only 46% of women and 26% of children report minimum dietary diversity and a minimum acceptable diet, respectively [8]. A national survey indicates that only 9% of children receive a minimum acceptable diet [7]. Given the large numbers of Tanzanian women less than 15 years of age, malnutrition will likely persist if the nutritional behaviors and practices of women of reproductive age remain sub-optimal.

Evidence suggests that activities such as dietary supplementation, food fortification and the promotion of dietary diversity improve maternal and child health outcomes [9]. Some studies indicate that having access to antenatal care services can positively influence birth outcomes including pre-term birth, birthweight, neonatal health, and the mother’s nutritional status [10–12], though several of these studies lack methodological rigor. Additional evidence suggests that providing nutrition education and counselling before and during pregnancy can improve maternal nutrition and neo-natal and child health [13–15]. As such, there is consensus that health clinics, community health volunteers, and community groups play an important role in the behavior change needed to improve health [14, 16, 17].

Even though the Tanzanian government provides free maternal and child health care services [18], access to services remains a problem [19]. Documenting women’s access to facility-based services and community outreach and understanding whether access to services is associated with women’s improved dietary practices are critical to designing and implementing effective policies and programs.

This research assesses whether women’s access to health services (through the government and through other implementing partners such as non-governmental organizations) is associated with dietary practices in pregnancy and post-partum. Specifically, we test the hypothesis that mothers with access to Tanzania’s existing health services are more likely than mothers without access to these services to eat more food, eat more frequently, and consume more diverse foods during pregnancy. We expect that these associations persist after adjusting for sociodemographic covariates.

## Methods

This study used data from a cross-sectional baseline survey using two-stage cluster sampling to randomly select respondents. The survey was conducted in January and February of 2016, prior to implementing a large, integrated nutrition project (Addressing Stunting in Tanzania Early or ASTUTE). In 2015, IMA (Interchurch Medical Assistance) World Health and its consortium partners launched a five-year projected funded by the United Kingdom Agency for International Development (UKAid). The project targeted women and children in five Northwestern regions of Tanzania, collectively representing a population of 10.2 million and more than 750,000 stunted children. These five regions were selected because they had a high prevalence of stunting and anemia and poor maternal and child feeding practices relative to the rest of the country. The baseline informed program design.

This study’s intent is to 1) document government and other implementing partner program coverage prior to the start of the ASTUTE project, and 2) assess associations between exposure to existing government programs and maternal health practices. After baseline, the ASTUTE project was implemented in five lake zone regions of Tanzania (Geita, Kagera, Kigoma, Mwanza, and Shinyanga) and its primary objective was to reduce stunting among children under 5 years of age with improvements in maternal diets, antenatal care seeking, hand hygiene, sanitation, and other behaviors as secondary objectives. Future research will examine associations between exposure to social and behavior change activities that were implemented as part of the ASTUTE project, antenatal care seeking, and maternal diet.

Study participants include 5000 female primary caregivers of children aged 0 to 23 months. We recruited respondents from five geographic regions, including Geita, Kagera, Kigoma, Mwanza, and Shinyanga. We used two-stage probability proportional to size sampling, first at the district level and then at the village level in rural areas and neighborhoods in urban areas, employing data from Tanzania’s most recent (2012) census as the sampling frame. Once randomly selected villages or neighborhoods were identified, we selected 20 households from within each village/neighborhood using a spin-the-bottle approach to choose an axis that interviewers could follow to identify the first household for interview. In rural areas, interviewers were required to identify houses at least 200 m apart. In urban areas, we selected every fifth house for interview (in buildings with more than one eligible household, only one household was interviewed).

We field-tested the survey instrument among mothers and fathers then revised and finalized it prior to administration by IPSOS (Institut de Publique Sondage d’Opinion Secteur) Tanzania, which is part of a global data collection firm. We scripted the questionnaire onto a mobile data collection platform and uploaded it to Android mobile devices used for data collection.

We obtained informed consent from all study participants—written if the respondent was literate and by thumb print if not. The National Institute for Medical Research in Tanzania and relevant local government authorities authorized the research (NIMR/HQ/R.8a/Vol.IX/2344). Three research teams, trained by IPSOS Tanzania, administered the questionnaire. Interviewers conducted one-hour face-to-face interviews in Kiswahili. Interviews took place at the caretaker’s place of residence. We made three attempts to contact mothers in their residence, after which replacement households were selected. There were 150 refusals total in the five regions (2.9% of all individuals contacted). Upon completion of data collection, IPSOS Tanzania compiled survey results for cleaning and analysis.

Outcomes included whether the mother, at any time during her most recent pregnancy resulting in the birth of the youngest living child, ate more than usual (a single, subjective measure) and consumed more types of foods than usual (also, a single, subjective measure). Additional outcomes included the number of times the mother ate in the previous 24 h and her dietary diversity based on seven food groups (grains, legumes and nuts, dairy, flesh foods, eggs, vitamin A rich foods, and other fruits), also measured for the 24 h prior to interview. Each of these behaviors was self-reported. Our measure of dietary diversity differs somewhat from the current 10-item Minimum Dietary Diversity for Women (MDD-W) index used by the Food and Agriculture Organization [20]. In particular, the MDD-W considers pulses (beans, peas and lentils) to be separate from nuts and seeds. The MDD-W also includes a category for other vegetables, in addition to dark, green leafy vegetables. However, when our baseline was carried out (2016), the Food and Agriculture Organization had not yet published new guidance about measuring maternal dietary diversity. Thus, our dietary diversity score is consistent with global standards as of early 2016.

Wherever possible, we used the same questions as those used in the 2015 Tanzania Demographic and Health Survey (TDHS) [7]. However, the TDHS does not ask about the amount and types of food consumed during pregnancy, exposure to counselling on maternal nutrition, contact with community health workers, nor participation in support groups. Each question not included in the TDHS was pre-tested then modified based on results from pre-testing.

Demographic data included information on the mother (ethnicity, religion, years of schooling, literacy, and age plus whether she personally owned a mobile phone), household (housing construction and assets ownership, whether the household owned a radio or TV, and the number of other children in the family), and community (travel time to the nearest market and health facility). The asset indicator was created by summing the number of assets respondents indicated they owned out of 13 possible assets, including bicycles, cars, carts, radio, and television, among others. The household construction index was created based on the construction materials used for the floor, roof, and walls of the dwelling ranging from three (if the walls, floor, and roof were made of rudimentary materials) to nine (if the walls, floor, and roof were made of finished materials). Access to services pertains to the availability of safe drinking water sources (e.g., protected wells, a public standpipe) and safe sanitation (e.g., a flush toilet). Pit latrines were not considered to be improved sanitation, per the Joint Monitoring Program of the WHO. The household wealth index was adapted from a previously validated index [21]. The index is comprised of the two sub-indices described above. An average of the two indices was used to calculate an overall wealth score, with possible values ranging between 0 and 1. Higher wealth scores indicate higher socioeconomic status.

Stata 14.2 (College Station, Texas, USA) was used for all statistical analyses. We calculated chi-squares and t-tests to gauge unadjusted associations between exposure to health programs and services and measures of maternal diet. Ordered logistic regression modeling was used to determine whether associations described in bivariate analysis persisted after adjusting for maternal age and education as well as household assets. These covariates were chosen based on conceptual and statistical considerations (including the need to avoid collinearity and overfitting models). Ordinary least squares modeling was conducted for outcomes that were continuous (mother’s dietary diversity and frequency of eating in the previous 24 h).

## Results

According to results presented in Table 1, most respondents were Christian, had a primary school education or higher, were literate, and lived a long distance from markets (27.8 min on average to reach the closest market) and health facilities (on average, a 33.9-min trip). About a third of the sample (34.1%) had received any antenatal care during their most recent pregnancy or had been advised from any source about nutrition when most recently pregnant (37.0%). During their most recent pregnancy, less than half (43.1%) of women received any advice whatsoever—regardless of source—about maternal nutrition. Of those receiving any advice, 93.5% were counselled by health facility or hospital staff, 0.9% by feeding center staff, 6.1% by CHWs, and 0.3% by pharmacists, and 68.0% had never had a CHW speak to them about their children’s health. Only one in ten (9.4%) of mothers had participated in a women’s group of any kind. Per self-reports, only 8.0% of mothers ate more than usual during their most recent pregnancy and 7.1% ate more types of foods. About a quarter of women (24.1%) consumed flesh foods in the previous 24 h while 1.2% had eaten eggs and 4.8% consumed vitamin A-rich foods (Table 1). A large percentage of mothers had not consumed meat (61.2%), fish (41.0%), eggs (89.5%), nor fruit (69.4%) in the previous week (results not shown in table form).

**Table 1:** Descriptive statistics for background variables, behavioral determinants, and maternal diet

	n	Mean or %
**Mother**		
Ethnicity (ref: Wasukuma)	5000	42.7
Wahaya		12.7
Waha		20.0
Other		24.6
Religion (ref: Christian Protestant)	5000	8.9
Christian, Catholic		43.5
Other Christian		41.7
Muslim		5.9
Years of schooling, mother (ref: no education)	5000	19.1
Primary incomplete		11.9
Primary complete		56.2
Secondary or more		12.9
Maternal literacy (ref: no)	5000	25.9
Yes		74.1
Maternal age (ref: 15–19 y)	4645	10.8
20–29 y		54.8
30–39 y		27.7
40–49 y		5.8
50+ y		0.9
Marital status		
Married monogamous		71.0
Married polygamous		5.8
Informal union		8.5
Other		14.7
Main occupation		
Crop farming		71.5
Self-employed		7.7
Housewife		15.1
Other		5.7
Mother personally owns a mobile phone	4997	40.8
**Household**		
Household construction (index: 3–9; mean)	5000	5.3
Household asset ownership (index 0–13; mean)	5000	4.2
Household has a radio	5000	47.5
Household has a TV	5000	11.7
Number of other children in the family (mean)	5000	1.6
**Community**		
Access to market (travel time, mean, in minutes)	5000	27.8
Access to health facility (travel time, mean, in minutes)	5000	33.9
**Access to health services**		
Mother received any antenatal care in most recent pregnancy^a^	4642	34.1
Mother received advice on maternal nutrition from any source before her pregnancy	4966	16.9
Mother received advice on maternal nutrition from any source during her pregnancy	4958	37.0
Mother received advice on maternal nutrition from any source after she gave birth to youngest child	4959	26.3
Times mother was exposed to nutrition counselling before and during pregnancy and after giving birth to youngest child		
0	4951	56.9
1		16.6
2		16.1
3		10.4
The last time a community health worker spoke with the mother about the child’s health generally		
Before or during most recent pregnancy^b^	5000	6.0
In the first few weeks and months after delivery	5000	12.6
Some other time	5000	13.4
No community health worker gave advice	5000	68.0
Mother participates in any women’s groups^c^	4995	9.4
**Access to land and fresh foods**		
Size of plot household cultivates for home garden and/or other agricultural land (mean, in acres)	5000	1.2 (95% CI 1.1 1.2)
Mother buys fresh foods daily or a few times a week	4986	67.3
**Mother’s dietary practices**		
Quantity of food mother ate during her most recent pregnancy	4645	
Less than usual		37.2
Same as usual		54.8
More than usual		8.0
Mother’s frequency of eating in previous 24 h	4972	
0–1 times		1.7
2 times		21.3
3 times		35.3
4 times		22.2
5 or more times		19.6
Mean		3.4 (95% CI 3.4, 3.5)
During her most recent pregnancy, mother ate:	4648	
Fewer types of foods		31.9
Same types of foods		61.0
More types of foods		7.1
Type of food mother consumed in previous 24 h		
Cereal	5000	95.0
Legumes and nuts	5000	36.0
Dairy	5000	2.5
Flesh foods	5000	24.1
Eggs	5000	1.2
Vitamin A-rich fruits	5000	4.8
Other fruits	5000	5.1
Mother’s dietary diversity in previous 24 h (7-item scale)	5000	
0 types of food		3.0
1 type of food		40.2
2 types of food		44.7
3 types of food		10.2
4 types of food		1.6
5 types of food		0.3
6 types of food		0.1
7 types of food		0.1
Mean		1.7 (95% CI 1.7, 1.7)

^a^Mothers rarely indicated another source of advice except for the health facility and CHW. 3.3% received information on maternal nutrition from their mother-in-law, 3.0% from a friend or neighbour, 1.3% from their husbands, and less than 1% from feeding centre staff, traditional healer, traditional birth attendant, pharmacist, religious leader, community leader, mother/mother-in-law, TV, radio, mobile phone, and print material.

^b^Includes 0.9% who indicated receiving advice before pregnancy.

^c^Less than 5% of women indicated they had participated in an agricultural group, a child health group, or a religious group.

Factors associated with eating more than usual during pregnancy included receiving any antenatal care, receiving advice about maternal nutrition during and after pregnancy (but not before), and speaking with a CHW about child health (Table 2). However, in many instances, these differences were small. For example, 9.2% of women who received any ANC during their most recent pregnancy and 7.3% of women who did not, ate more than usual during pregnancy. Receiving any ANC; being counselled about nutrition before, during and after pregnancy; being advised at least once about nutrition; and speaking with a CHW about child health generally were associated with eating more types of foods during pregnancy. All program exposure variables were significantly associated with mothers’ frequency of eating in the previous 24 h (*p* < 0.001 for all comparisons). Mothers’ dietary diversity over the same time period was associated with receiving any ANC, receiving advice about maternal nutrition during and after pregnancy (but not before), and speaking with a CHW about their child’s health.

**Table 2:** Associations between health services and food consumption during pregnancy and in the previous 24 h

Recall period:	Most recent pregnancy				Last 24 h			
Variable	Mother ate more than usual during pregnancy		Mother ate more types of foods		Mother’s frequency of eating		Dietary diversity	
	%	*P*	%	*P*	Mean	*P*	Mean	*P*
Mother received any ANC in the most recent pregnancy								
Yes	9.2	0.019	9.9	< 0.001	3.7	< 0.001	1.7	0.004
No	7.3		5.7		3.3		1.7	
Mother received advice on maternal nutrition from any source before her pregnancy								
Yes	8.7	0.424	11.2	< 0.001	3.8	< 0.001	1.7	0.226
No	7.8		6.3		3.4		1.7	
Mother received advice on maternal nutrition from any source during her pregnancy								
Yes	9.7	0.001	9.6	< 0.001	3.6	< 0.001	1.8	< 0.001
No	6.9		5.7		3.4		1.6	
Mother received advice on maternal nutrition from any source after she gave birth to youngest child								
Yes	9.4	0.038	9.3	0.001	3.6	< 0.001	1.8	< 0.001
No	7.5		6.4		3.4		1.6	
Mother was advised at least once about nutrition before and during pregnancy and after giving birth to youngest child								
Yes	9.5	0.001	9.1	< 0.001	3.5	< 0.001	1.8	< 0.001
No	6.8		5.6		3.3		1.6	
CHW spoke with mother about child health generally								
Yes, advice at any time	9.7	0.004	8.3	0.016	3.6	< 0.001	1.8	< 0.001
No	7.2		6.4		3.3		1.6	
Mother participates in any women’s groups								
Yes	7.6	0.789	7.6	0.674	3.6	< 0.001	1.7	0.127
No	8.0		7.1		3.4		1.7	

Results from ordered logistic regression models that adjusted for mother’s age, education and household assets (Table 3) suggest that only one variable (receiving advice on nutrition during pregnancy) was associated with the mother eating more food during her most recent pregnancy. Women who received advice were 1.3 times (95% CI: 1.1, 1.7) more likely than mothers who did not receive advice to eat more during pregnancy. Women who received ANC and advice on nutrition before, during, and after pregnancy and birth were all significantly more likely than women who did not to eat more types of foods. Hearing from a CHW about their child’s health and participating in support groups were largely not associated with eating more types of foods.

**Table 3:** Ordered logistic regression: Association between development assistance, health services, and mother’s dietary practices

	Mother ate more food during her most recent pregnancy			Mother ate more types of foods during her most recent pregnancy		
	AOR	95% CI	*P*	AOR	95% CI	*P*
**Access to health services**						
Mother received any antenatal care in most recent pregnancy	1.2	1.0, 1.5	0.111	1.7	1.3, 2.1	< 0.001
Mother received advice on maternal nutrition from any source before her pregnancy	1.1	0.8, 1.4	0.730	1.8	1.4, 2.3	< 0.001
Mother received advice on maternal nutrition from any source during her pregnancy	1.3	1.1, 1.7	0.010	1.7	1.3, 2.1	< 0.001
Mother received advice on maternal nutrition from any source after she gave birth to youngest child	1.2	1.0, 1.5	0.102	1.4	1.1, 1.8	0.003
The last time a community health worker spoke with the mother about the child’s health generally						
No community health worker gave advice (ref)						
Before most recent pregnancy	2.2	0.9, 5.4	0.078	0.7	0.2, 2.9	0.631
During most recent pregnancy	1.1	0.7, 1.9	0.585	1.1	0.6, 1.8	0.778
In the first few weeks and months after delivery	1.2	0.9, 1.7	0.260	1.5	1.1, 2.0	0.015
Some other time	1.3	1.0, 1.8	0.066	2.0	0.9, 1.7	0.293
Mother participates in any women’s groups	1.0	0.7, 1.4	0.904	1.0	0.7, 1.5	0.810

Note: Adjusted for mother’s age and education and household assets

According to results from ordinary least squares models (Table 4), women who received advice about maternal nutrition during and after pregnancy, who had heard CHWs’ advice about child health generally, and who had participated in any women’s group were significantly (*p* < 0.05) more likely than women who had not to eat more frequently in the previous 24 h. Additionally, mothers who received ANC care; those who received advice about maternal nutrition from any source before, during, and after pregnancy; and mothers hearing from CHWs during and after delivery about the child’s health were generally more likely than those who did not receive care nor counselling to consume a more diverse diet in the previous 24 h, after adjusting for mother’s age and education and household assets (*p* < 0.01 for all comparisons found to be significant).

**Table 4:** Ordinary least squares: Associations between access to development assistance, health services, and mother’s dietary diversity

	Mother’s frequency of eating in previous 24 h			Mother’s dietary diversity in previous 24 h, 7-item scale		
	Coefficient	95% CI	*p*	Coefficient	95% CI	*p*
**Access to health services**						
Mother received any antenatal care in most recent pregnancy	0.2350	0.1697, 0.3003	< 0.001	0.0179	−0.0321, − 0.0679	0.484
Mother received advice on maternal nutrition from any source before her pregnancy	0.3426	0.2633, 0.4220	< 0.001	−0.0258	−0.0866, 0.0351	0.406
Mother received advice on maternal nutrition from any source during her pregnancy	0.0959	0.0330, 0.1588	0.003	0.0722	0.0243, 0.1201	0.003
Mother received advice on maternal nutrition from any source after she gave birth to youngest child	0.1888	0.1205, 0.2571	< 0.001	0.1094	0.0573, 0.1615	< 0.001
The last time a community health worker spoke with the mother about the child’s health generally						
No community health worker gave advice (ref)						
Before most recent pregnancy	0.2232	−0.0837, 0.5301	0.154	−0.0238	−0.2563, 0.2087	0.841
During most recent pregnancy	0.4812	0.3450, 0.6173	< 0.001	0.3133	0.2090, 0.4175	< 0.001
In the first few weeks and months after delivery	0.2536	0.1627, 0.3445	< 0.001	0.0421	−0.0273, 0.1112	0.235
Some other time	0.0534	−0.0365, 0.1432	0.244	0.0856	0.0169, 0.1543	0.015
Mother participates in any women’s groups	0.1594	0.0566, 0.2623	0.002	0.0256	−0.0528, 0.1041	0.522

Note: Adjusted for mother’s age and education and household assets

## Discussion

This paper examined potential associations between existing access to health services and other sources of advice about nutrition and women’s dietary practices during pregnancy and post-partum. We found that only 34% of women received any ANC, a sharp contrast to national figures which indicate that 98% of all pregnant women receive at least some antenatal care [7]. Consistent with study hypotheses, we found that mothers who had made at least one ANC visit, had heard advice about their own nutrition and the health of their child, and to a lesser extent, had benefited from visits by CHWs, were more likely to consume a diverse diet. These findings are largely consistent with the existing literature. Gludeirard and Olude [22] note that nutrition education and counselling have mostly been associated with improved maternal dietary practices. In India, Ghosh-Jerath and colleagues [23] found that ANC visits were associated with protein uptake. In Nepal, Sunuwar and collaborators [24] documented significantly higher intake of red meat, fish and liver, vitamin C-rich fruits, dairy products, eggs, and green leafy vegetables among women making ANC visits. In Egypt and in Senegal, exposure to positive deviance programs was associated with consuming more meat and vegetables [25] and iron supplements [26]. In a large study from Bangladesh, relative to a standard maternal, neonatal, and child health package, women who received intensive nutrition counselling via salaried health workers and incentivized community health volunteers, monthly home visits, one-on-one ANC sessions with individually-tailored diet plans, and nutrition promotion activities among family members were more likely to consume a greater number of food groups, specific foods, and iron and calcium supplements [27]. In contrast, some studies, including one from Nepal [28], report that women participating in support groups did not have significantly more diverse diets nor protein and energy adequacy than non-participants.

We also found that mothers who received any ANC during pregnancy ate more frequently, compared to women who did not receive such services. There is considerably less literature on nutrition promotion and feeding frequency but Demilew and colleagues [29] and Diddana [30] report from their observational study in Ethiopia that knowledge about diet during pregnancy was associated with consuming foods more frequently. Our findings indicate that mothers who received advice on maternal nutrition from any source during pregnancy self-reported eating more food compared to mothers who did not receive such advice, a finding noted by Ahrari and colleagues [25] in Egypt and Nguyen and colleagues [27] in Bangladesh.

In addition to program exposure, other factors are known to influence nutrition behaviors. These include women’s knowledge of nutrition, perceived severity of malnutrition, poor perceived benefits of adopting healthy nutrition practices, and low self-efficacy [29–31].

Findings reported here demonstrate at least modest associations between access to health facility and community-based services and a range of nutrition-related behaviors. However, only a third of our sample received any ANC during their most recent pregnancy and a similarly low percent received nutrition advice from any source. Less than half of women received any advice on maternal nutrition—professional or otherwise--during their most recent pregnancy and for those who had been advised, the majority of that advice occurred in hospitals or health facilities, with only about 5% coming from CHWs. Similarly, just one in ten mothers had participated in a women’s group of any kind—a potential forum for discussing women’s nutrition. Other studies in Tanzania also report low exposure to facility-based counselling and frontline health worker activities [7, 32]. While our results regarding the potential impact of ANC and CHW counselling are encouraging, population-level changes in nutrition behaviors are unlikely to occur at the scale reported in Bangladesh and elsewhere [28], barring major efforts to scale up current interventions. Nguyen and colleagues note that in Bangladesh, among seven program elements, differences in intervention coverage were one of two factors that best explained the lack of program impact [27].

Achieving scale is only one potential challenge to improving nutrition behaviors. Others include health worker shortages such as a lack of dieticians [33], low salaries [34], health workers’ shortage of time [28], inadequate information and training to counsel women [13, 33–35], and uncertainty about how to translate general nutrition requirements to individual needs [13].

We used project baseline data from a large survey to explore the relationship between exposure to existing government programs and dietary practices among women in a specific geographic region of Tanzania. Our findings provide useful information to policy makers and program planners who are tasked with improving women’s nutrition. However, our study has limitations. These include single, subjective measures to gauge two variables “ate more than usual” and “consumed more types of foods than usual,” given that it was not possible to quantify the actual amount of food women consumed before giving birth. Twenty-four-hour dietary recall provided a more objective measure of dietary diversity but was measured in the 24 h prior to interview, not during women’s pregnancy. Other limitations included lack of more detailed measures of the quality, timing, and nature of programs and source of nutrition information (for example, clinicians versus CHWs versus family members or friends). Additionally, data are cross-sectional, thus restricting our ability to infer causality, and only include information on exposure to existing government services, thus limiting our ability to document changes in program exposure and nutritional practices over time. Further analyses based on the ASTUTE project’s midline and endline will shed light on apparent anomalies in our findings, for example, that counseling after pregnancy was associated with eating more foods and more types of foods during pregnancy.

A number of factors influence women’s diets, including production of nutritious, diverse foods; women’s position within the household, including control over resources and decision-making power; how foods that the household uses are produced; what crops are designated for home consumption; cultural beliefs about what foods women should eat during pregnancy and lactation; and the emotional and instrumental support other family members offer to promote dietary behaviors known to improve women’s nutrition [8, 36]. Programs designed to improve women’s dietary behaviors can benefit from efforts to bolster both the number and preparedness of health workers (including training to standardize innovative counselling and community-based behavior change strategies), a re-structuring of salaries and incentives for health workers, greater time allocation for nutrition education and counselling, and more supportive supervision. Standardization of roles and responsibilities, job aids, and frequent monitoring of nutrition education and counselling are critical to implementing high-quality interventions.

Future research would benefit from elucidating how the timing, content, and source of counselling and community mobilization impact upon women’s dietary behaviors [16, 37]. Intervention research that more fully characterizes programs [35] and elucidates how and why interventions achieve impact will benefit policy and program design and implementation. Rigorous program fidelity assessments can pinpoint which of various program elements might explain the impact of a given intervention.

## Conclusions

Consistent with much of the existing literature, our research reinforces the importance of receiving advice about nutrition and health from health facility workers and frontline health workers/volunteers before and during pregnancy as well as post-partum. Given the impact of nutrition education and counselling on women’s diets, it is essential that future attempts to improve women’s dietary behaviors consider the complex environment within which women operate. Improving mothers’ diets should include training and supportive supervisory systems, the introduction of proven counselling and behavior change strategies, community social mobilization and greater political will to extend health facility and community-based outreach.

## Data Availability

The datasets generated and analyzed during the current study are not publicly available due to concerns about the confidentiality of individual respondents but are available from the corresponding author on reasonable request.

## Abbreviations

ANC: Antenatal care
ASTUTE: Addressing stunting early in Tanzania
BMI: Body Mass Index
CHW: Community health worker
IMA: Interchurch Medical Assistance
IPSOS: Institut de Publique Sondage d’Opinion Secteur
MDD-W: Minimum Dietary Diversity for Women
LMICs: Low- and middle-income countries
TDHS: Tanzanian Demographic and Health Survey
UKAid: United Kingdom Agency for International Development
